# Periapical bacterial disinfection is critical for dental pulp regenerative cell therapy in apical periodontitis in dogs

**DOI:** 10.1186/s13287-023-03628-6

**Published:** 2024-01-17

**Authors:** Koichiro Iohara, Michiyo Tominaga, Hideto Watanabe, Misako Nakashima

**Affiliations:** 1 Section of Regenerative Dental Medicine, National Center for Geriatrics and Gerontology, Research Institute, Geroscience Research Center, 7-430 Morioka, Obu, Aichi 474-8511 Japan; 2 Institute for Molecular Science of Medicine, Aichi Medical University, Nagakute, Aichi 480-1195 Japan; 3 Air Water Group, Aeras Bio Inc., Kobe, Hyogo 650-047 Japan

**Keywords:** Pulp regeneration apical, Periodontitis, Dental pulp stem cells, Allogeneic cell transplantation, Periradicular disinfection

## Abstract

**Background:**

Application of pulp regenerative cell therapy for mature teeth with periapical lesions is a critical clinical challenge. The bacterial infection in inaccessible location within the root canal system and in the periapical lesions could cause resistance and impediment, leading to limitations in successful therapy. Thus, the aim of this study was to examine the effect of residual bacteria on the outcome of pulp regeneration in mature teeth with apical periodontitis in dogs.

**Methods:**

Periapical lesions were induced in 32 root canals of 4 dogs in two different models in severities, model A and model B. Model A (moderate infection): the canal exposed to the oral cavity for 2 weeks and then closed for 2 weeks. Model B (severe infection): the canal exposed to the oral cavity for 2 months and then closed for 5 months. All root canals were irrigated with 6% sodium hypochlorite, and 3% EDTA and further with 0.015% levofloxacin-containing nanobubbles, which was also used as an intracanal medicament. The aseptic conditions were examined by bacterial anaerobic culture and/or PCR analyses. The root canal treatment was repeated several times, and allogeneic dental pulp stem cells were transplanted into the root canals. The radiographic evaluation of periapical lesions was performed by cone-beam computed tomography before the first treatment, just after cell transplantation, and after 2 months and 6 months in both model A, model B, respectively. The animals were then sacrificed and the jaw blocks were harvested for histological and histobacteriological evaluations of pulp regeneration and periapical tissue healing. Furthermore, the DiI-labelled DPSCs were transplanted into the root canals after complete disinfection (*n* = 4) or without root canal treatment (*n* = 4) in the apical periodontitis model (model A) in one dog, and cell localization was compared 72 h after transplantation.

**Results:**

In 8 out of 12 canals from model A, and 10 out of 15 canals from model B, pulp regeneration with good vascularization, innervation, and a significant reduction in the radiolucent area of the periapical lesions were observed. However, in the other 4 canals and 5 canals from model A and model B, respectively, no pulp tissue was regenerated, and inflammation in the periapical tissue, and external resorption or healed external resorption were detected. The presence of residual bacteria in the periapical tissues and severe inflammation were significantly associated with inhibition of regenerated pulp tissue in these 9 unsuccessful canals (*P* < 0.05, each) (OR = 0.075, each) analyzed by multiple logistic regression analysis. For cellular kinetics, transplanted cells remained in the disinfected root canals, while they were not detected in the infected root canals, suggesting their migration through the apical foramen under the influence of inflammation.

**Conclusions:**

A true pulp-dentin complex was regenerated in the root canal by the pulp regenerative therapy in mature teeth with apical lesions. The successful pulp regeneration was negatively associated both with residual bacteria and inflammation in the periapical tissue.

**Supplementary Information:**

The online version contains supplementary material available at 10.1186/s13287-023-03628-6.

## Background

Clinical regenerative cell therapy of pulp (cell-based regenerative endodontics, CB-RET [1]) using dental pulp stem cells (DPSCs) in mature teeth has been successfully performed for irreversible pulpitis [2–4]. However, in an aging society, such as in Japan, CB-RET in mature teeth must be examined also for apical periodontitis to reestablish tooth vitality and function, leading to endurance of the teeth.

As for mature necrotic teeth with apical periodontitis, a systemic review and meta-analysis of 4 randomized control trials recently demonstrated that cell-free regenerative endodontic therapy (CF-RET) (revitalization/revascularization) [5] via blood clot appears a viable treatment alternative to traditional endodontic procedures [6]. Successful treatment outcomes in these clinical trials, however, were based on the resolution of clinical symptoms and radiographic healing of periapical lesions [7, 8]. Arslan et al. [7] reported that half of the mature teeth with apical periodontitis after CF-RET showed a positive response to electric pulp testing (EPT) as the secondary outcome. El-Kateb et al. [9] also reported that in mature teeth 12 months after CF-RET, MRI signal intensities of the root canals were similar to those of the normal pulp of the contralateral tooth, and approximately more than 60% of all treated teeth responded positively to the EPT. However, EPT and MRI do not necessarily indicate the regeneration of organized pulp-dentin complex in the mature teeth. A histological analysis of the regenerated tissue was performed in a mature tooth that was clinically and radiographically deemed healed by CF-RET and had a horizontal crown fracture later. It showed repair by fibrous connective tissue including periodontal ligament, cementum, and bone-like tissues, and inflammation [7], similarly as in previously reported immature teeth [1].

The major goals of CB-RET in mature teeth with apical periodontitis are not only to encourage periapical tissue healing, but also to regenerate true pulp-dentin complex in the root canal. This could be accompanied by reinnervation, revascularization, normal homeostatic function, capability of the dentin formation along the lateral wall and dentin bridge formation, and immunomodulatory function, which may make the tooth resistant to bacterial infection and fracture. In immature teeth with necrotic/apical periodontitis, a clinical trial of CB-RET using autologous DPSCs was performed for treating tooth injuries. A histological and immunohistochemical analysis of retraumatized human tooth at 12 months after the CB-RET showed successful regeneration of whole pulp tissue containing blood vessels, nerve generation and an odontoblastic layer [10]. While in mature teeth with apical periodontitis the efficacy of CB-RET was demonstrated using allogeneic MSCs (umbilical cord mesenchymal stem cells) in a clinical trial only based on clinical and radiographic healing as a primary outcome. The percentage of perfusion unit between baseline and after 12 months was increased by laser Doppler flowmetry. A positive response to cold and electric tests (56% and 50%, respectively), and the remission of apical lesions were demonstrated. Those findings could be used as an indicative of the formation of a vascularized tissue with a normal physiological response, suggesting probably vital pulp-like tissue [11]. Histological evaluation of regenerated tissues in the root canal spaces after CB-RET for mature teeth with apical periodontitis, however, has not been performed in human. In experimental animal models, histological evaluations were performed only in immature teeth [12–14] and has not been reported in mature teeth.

Endodontic treatments have a higher chance of failure if microorganisms persist in the root canals at the time of obturation [15]. Bacteria harbored in the root canal system, such as isthmuses, dentinal tubules, and ramifications, may evade disinfectants and persist getting the unchanged nutrient supply [16]. The presence of residual bacteria was significantly associated with lack of root development and less closure of open apex in CB-RET for immature ferret teeth with periapical lesions [12]. A study demonstrated that root canals with residual bacterial levels below 3 × 10^3^ cells upon obturation had a satisfactory outcome [17]. However, conventional disinfection protocols are ineffective at reducing cell numbers to less than 3-log. It is necessary for pulp regeneration to be a 5-log reduction in bacterial number (99.999%) in the root canal microenvironment [18]. Furthermore, when pathogens are removed from the root canal, conventional irrigation and medicaments also can kill host stem cells that reside in the apical tissue, inhibiting their migration and repopulation in the root canal [18]. We have successfully used antibiotics containing nanobubbles for irrigation and as intracanal medicaments in addition to conventional irrigation in initial root canal treatment without apical lesions [2]. Shawli et al. reported that nanobubbles have the potential to remove the smear layer and enhance tubular penetration of medicament [19]. The effects of this disinfection protocol and the resulting residual bacteria on pulp regeneration in mature teeth with apical periodontitis, however, have not been elucidated. Thus, we must examine the aseptic conditions. For confirming the aseptic conditions, we added PCR assay which is more useful for detecting uncultured bacteria [20] to a bacterial anaerobic culture.

The purpose of this study is to histologically analyze the results of pulp regeneration with DPSCs in a canine model of apical periodontitis both with moderate and severe infection, and to investigate the effect of residual bacteria on pulp regeneration and healing of periapical lesions by histobacteriological and radiological analyses. In addition, cellular kinetics of transplanted DPSCs was examined in the infected teeth with or without disinfection to further elucidate the effect of residual bacteria on pulp regeneration. For the first time, this study provided histological evidence in the animal experiments to support the application of CB-RET with allogeneic DPSCs for the treatment of mature teeth with apical periodontitis after appropriate disinfection of infected root canal systems.

## Materials and methods

All animal procedures were approved by the Animal Care and Use Committee of the National Center for Geriatrics and Gerontology, Research Institute (permission #2-17, #3-47, #4-43) and Aichi Medical University (permission #2020-93, #2021-85, #2022-57).

### Cell isolation and cell culture

When canine experiments were conducted, an initial intramuscular injection of atropine sulfate (Mitsubishi Tanabe Pharma, Osaka, Japan) was administered at 0.04 mg/kg as premedication, followed by an intramuscular injection of a three-mixture solution to induce anesthesia. This mixture included 0.2 mg/kg of butorphanol tartrate (Meiji Seika Pharma, Tokyo, Japan), 80 µg/kg of medetomidine hydrochloride (Nippon Zenyaku Kogyo, Fukushima, Japan), and 0.1 mg/kg of midazolam (Astellas Pharma, Tokyo, Japan). Under local anesthesia with lidocaine (Dentsply Sirona, Tokyo, Japan), upper canine teeth were extracted from 9-month-old female dogs (*n* = 3) (Kitayama Labes, Ina, Japan). DPSCs were isolated and expanded in T-flasks at 3% oxygen concentration as described previously [21] with a slight modification of not rotating the culture at the third passage. DPSCs were cryopreserved at 1 × 10^6^ cells/mL at the 4th passage for allogeneic transplantation.

### Apical periodontitis models in dogs

After pulp extirpation, a total of 32 root canals of the upper 2nd and lower 3rd premolars from four 12–13-month-old female dogs were enlarged to #55 using a K-file (MANI, Utsunomiya, Japan). Two models of periapical lesion infection severity (Model A, Model B) were induced in 2 dogs each. Model A (moderate infection, easy to disinfect): the canal exposed to the oral cavity for 2 weeks and then sealed for 2 weeks with hydraulic cement (GC, Tokyo, Japan) and composite resin (Kurare Noritake, Tokyo, Japan). Model B (severe infection, refractory to disinfect): the canal exposed to the oral cavity for 2 months and then sealed for 5 months. Development of periapical lesions were confirmed using a Veraviewepocs 3D cone-beam computed tomographer (CBCT) (Morita Group, Suita, Japan). Nanobubbles (Aeras Bio Inc. Kobe, Japan) was placed in each root canal and allowed to stand for 2 min. A sterile paper point was inserted into each root canal for 1 min to collect a sample, and was kept in 250 µL of liquid culture medium (PLADIA (Showa Yakuhin Kako Co., LTD, Tokyo, Japan) or in GAM Broth, modified Nissui (Nissui Pharma Solutions, Shimadzu Diagnostics Corp., Tokyo, Japan) for several hours under anaerobic conditions using a deoxygenating agent (Mitsubishi Gas Chemical Company, Inc., Tokyo, Japan). Subsequently, 200 μL of each culture medium sample was plated on Blood Agar (BD Japan, Tokyo, Japan) using serial dilution. The number of colonies was counted after 5 days of anaerobic culture. Each volume of the radiolucent area and the assumed periapical lesions in CBCT images were analyzed by OsiriX medical imaging software (www.osirix-viewer.com), a DICOM viewer program. Any correlation between the number of harvested bacteria in the root canals and the volume of periapical lesions before root canal treatment was examined.

For DNA extraction, samples isolated from each bacterial colony each from model A and model B in microtubes containing 50 μL of liquid culture media were vortex for 1 min to disperse the microbial suspensions. The suspensions were centrifuged at 3,000 rpm for 10 min and the supernatant was removed. Each residue was suspended in 5 μL of sterilized distilled water, added 5 μL of 100 mM NaOH and incubated at 95 °C for 15 min after vortex. Finally, 1.5 μL of 1 M Tris–HCl (pH 7.0) was mixed and then used as the DNA sample. Those samples were analyzed by PCR assay using universal genes to amplify a specific region (approx. 0.8 kb) within bacterial 16S rDNA. The PCR reaction mixture had 25 μL that contained 12.5 μL of TaKaRa Taq™ HS Fast Detect Premix (2 ×) (Takara Bio Inc., Kusatsu, Japan), 2.5 μL of 16S rDNA Primer mix (800) (10 ×) (containing Sequencing Primer 10F, and Sequencing Primer 800R) (Bacterial 16S rDNA PCR Kit Fast (800), Takara Bio Inc.), 2.5 μL of the extracted DNA and 2.5 μL of PCR water. A known bacterial isolate (*E. coli*) and PCR water were used as a positive control and a negative control, respectively. PCR reactions were performed in Applied Biosystems 7500 Real-Time PCR at conditions at 92 °C for 5 s; 50 °C for 1 s; 68 °C for 8 s for 27 cycles, held at 4 °C, and PCR products were stored at − 20 °C until gel electrophoretic analyses. Furthermore, 16S rRNA amplicon sequencing were performed using the PCR products using the forward primer (Sequencing Primer 10F), and the reverse primer (Sequencing Primer 800R) (commissioned to Takara Bio, Inc.), and microbiomes were identified by NCBI BLAST.

### Bacterial susceptibility test

*Fusobacterium nucleatum* (ATCC 25586), *Enterococcus faecalis* (ATCC 19433), and harvested samples from root canals that developed periapical lesions were amplified by anaerobic culture in brain heart infusion (BHI) medium (Kanto Chemical Co., Inc., Tokyo, Japan) for 24 h, then plated on BHI agar for antibacterial susceptibility tests. Three minutes after plating, disks absorbed with 0.0015%, 0.015%, 0.15%, and 1.5% levofloxacin (Santen, Osaka) were placed on top of the BHI agar and incubated at 37 °C for 24 h. The inhibition circles were used to determine microbe sensitivity to levofloxacin.

### Root canal treatment

The root canals were irrigated with 6% sodium hypochlorite (Yoshida Pharmaceutical, Saitama, Japan), 3% EDTA (Smear Clean^®^, Nippon Shika Yakuhin Co., Ltd. Shimonoseki, Japan) and saline solution (Otsuka Pharmaceutical, Tokyo, Japan), followed by nanobubbles (Aeras Bio Inc., Kobe, Japan, 1.0–1.4 × 10^8^/ml in concentration, − 20 to − 30 mV in zeta potential, 100–150 nm in diameter) for 3 min. Afterward, the root canals were thoroughly irrigated with 0.015% levofloxacin-containing nanobubbles, and then dried. Paper points soaked with levofloxacin-containing nanobubbles were inserted into the root canals as an intracanal medicament and temporarily sealed with hydraulic cement and composite resin. One week later, the aseptic conditions of each root canal were examined by colony counting 5 days after bacterial anaerobic culture in BHI medium and/or PCR assays as described above. They were then disinfected similarly without irrigation of 3% EDTA. This root canal treatment was repeated prior to cell transplantation.

### DPSC transplantation

After examining the number of remaining bacteria in the root canals with periapical lesion in model A (*n* = 12) by colony counting and in model B (*n* = 15) by colony counting and PCR assay, pulp regenerative cell therapy was performed as described previously [22]. In brief, the root canals were irrigated as described above, and further with 3% EDTA for 3 min, then with saline and dried well. Allogeneic DPSCs (5 × 10^5^ cells) were suspended in 20 μL of collagen (Koken, Tokyo, Japan) and 150 ng of G-CSF (Chugai Pharmaceutical Co. Ltd., Tokyo, Japan) and were transplanted into the root canals. The cavity was sealed with cement (Biodentine, Septodont, St. Maur-des-Fossés, France) and a composite resin. All teeth were imaged using CBCT immediately after, and 2 months (model A) and 6 months (model B) after transplantation before extraction.

### Histological and histobacteriological analyses of regenerated tissues

Before extraction, the dogs were sacrificed under general anesthesia (sodium pentobarbital: 200 mg/kg i.v.). All extracted teeth, including the periapical tissue, were morphologically examined in 5-μm paraffin sections using hematoxylin and eosin staining. To examine the relative amount of regenerated pulp tissue to the root canal space, five sections every 25 μm-interval were taken from each sample for a total of 8 samples from model A and 10 samples from model B. Immunostainings with BS-1 lectin (Vector Laboratory, Newark, California), PGP9.5 antibody (UltraClone, Cambridge, U.K.), and DSPP antibody (Santa Cruz Biotechnology, Dallas, Texas) were performed to examine neovascularization, reinnervation, and odontoblastic differentiation, respectively, in the regenerated tissue. Gram staining (Merck, Darmstadt, Germany) was performed to localize residual bacteria.

The root canals were categorized into two groups based on pulp regeneration, the success group (*n* = 8, and *n* = 10, in model A, and model B, respectively) and the failure group (*n* = 4, and *n* = 5, in model A, and model B, respectively). The success group contained the root canals which relative amount of regenerated pulp was more than 30%. The presence of internal and external resorption, and the presence of residual bacteria were compared between the two groups in both models by counting the score 1 for presence and the score 0 for absence. The degree of inflammation in the periapical tissue was also compared by the Dental Apical Inflammation Score (DAIS) (1: low acute inflammation and low chronic inflammation, 2: low acute inflammation and high chronic inflammation, 3: high acute inflammation and low chronic inflammation, 4: high acute inflammation and high chronic inflammation) [23].

### Analyses of apical lesion volume by CBCT images

Serial changes in apical lesion volume were examined by CBCT images using OsiriX medical imaging software before starting root canal treatment, at transplantation and at extraction (2 months after transplantation in model A, and 6 months after transplantation in model B) in the two groups in model A and model B. In the success group in model A and model B, associations of relative amount of regenerated pulp with harvested bacterial numbers and with lesion volumes at before starting root canal treatment were examined, respectively. Furthermore, associations of relative amount of regenerated pulp with reduced rates of lesion volume from pre-treatment to transplantation, from pre-treatment to extraction, from transplantation to extraction were further calculated.

### Cellular kinetics

The DiI (Invitrogen, Carlsbad, CA)-labelled DPSCs were transplanted into the root canals after complete disinfection (*n* = 4) or without root canal treatment (*n* = 4) in the upper 2nd and lower 3rd premolars of the apical periodontitis model in one 20-month-old dog. Cell localization was compared 72 h after transplantation between the two groups, assuming that chronic inflammation due to intracanal and periradicular bacteria could remain in the apical periodontitis model without root canal treatment.

### Statistical analysis

Statistical analyses were performed using the Statistical package for Social Sciences (SPSS), version 25.0 (IBM, Armonk, NY). All values are expressed as the mean ± SD. The *P* value for comparison of two groups was derived from the independent-samples *t* test. The correlation analysis was performed by the Pearson correlation test. The correlations of the success of pulp regeneration with the presence of morphologically evident remaining bacteria in periapical tissue, and with inflammation, internal resorption, or external resorption were analyzed using multiple logistic regression analysis.

## Results

### Apical periodontitis models

**Fig. 1:**
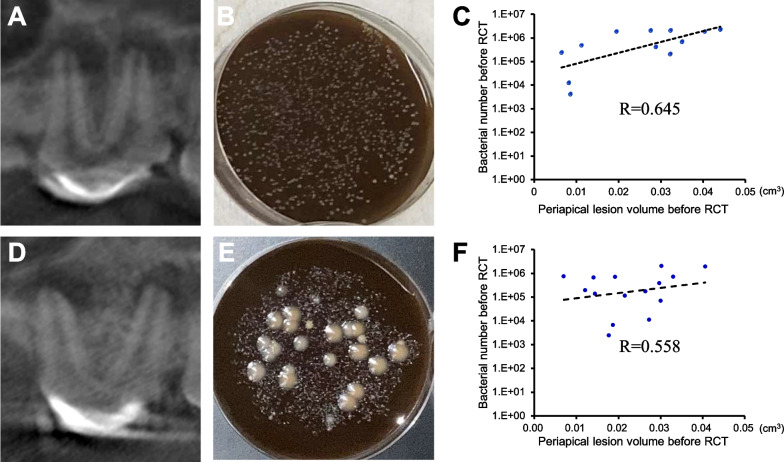
Establishment of apical periodontitis models in dogs. **A**–**C** Model A. **D**–**F** Model B. **A**, **D** Periapical lesions captured by cone-beam computed tomography (CBCT). **B**, **E** Bacterial colonies in brain heart infusion agar 48 h after plating. **C**, **F** Correlations of periapical lesion volume to bacterial numbers before root canal treatment (C: *R* = 0.645, F: *R* = 0.558)

CBCT image showed apical lesions (Fig. 1A, D) in 12 out of 16 root canals in model A and 15 out of 16 root canals in model B, establishing apical periodontitis models. In model B, swelling and fistulae were found in 6 out of the 15 root canals and CBCT showed root resorption in 10 out of the 15 root canals. Bacteria harvested from the root canals before the start of treatment showed several distinct types of colonies in sizes and colors after 72 h-cultivation (Fig. 1B, E). The bacteria from the various colonies were identified as *Lactobacillus murinus*, *Escherichia coli strai*, *Tissierella sp*, *Fusobacterium sp,* and *Streptococcus gordonii* in model A, and *Enterococcus faecalis, Porphyromonas sp*, *Prevotella sp*, *Streptomyces sp*, *Tannerella forsythia*, *Moraxella osloensis*, *Tessaracoccus massiliensis*, *Micrococcus luteus*, *Macrococcus carouselius* and *Neisseria zoodegmatis* in model B.

The lesion volumes of CBCT image before starting root canal treatment were not significantly different between model A and model B (25.3 mm^3^ ± 12.7 (*n* = 12) and 24.6 mm^3^ ± 10.0 (*n* = 15), respectively, *P* = 0.431). However, bacterial numbers and lesion volume were highly related both in model A and model B (*R* = 0.645, *R* = 0.558, respectively) (Fig. 1C, F).

In bacterial susceptibility testing, inhibition circles by levofloxacin were formed at the concentration higher than 0.015% for *F. nucleatum*, *E. faecalis* and bacteria isolated from model A and B, suggesting a sensitivity of these bacteria to levofloxacin (Data not shown).

### Histological analysis of regenerated tissues in the success groups

**Fig. 2:**
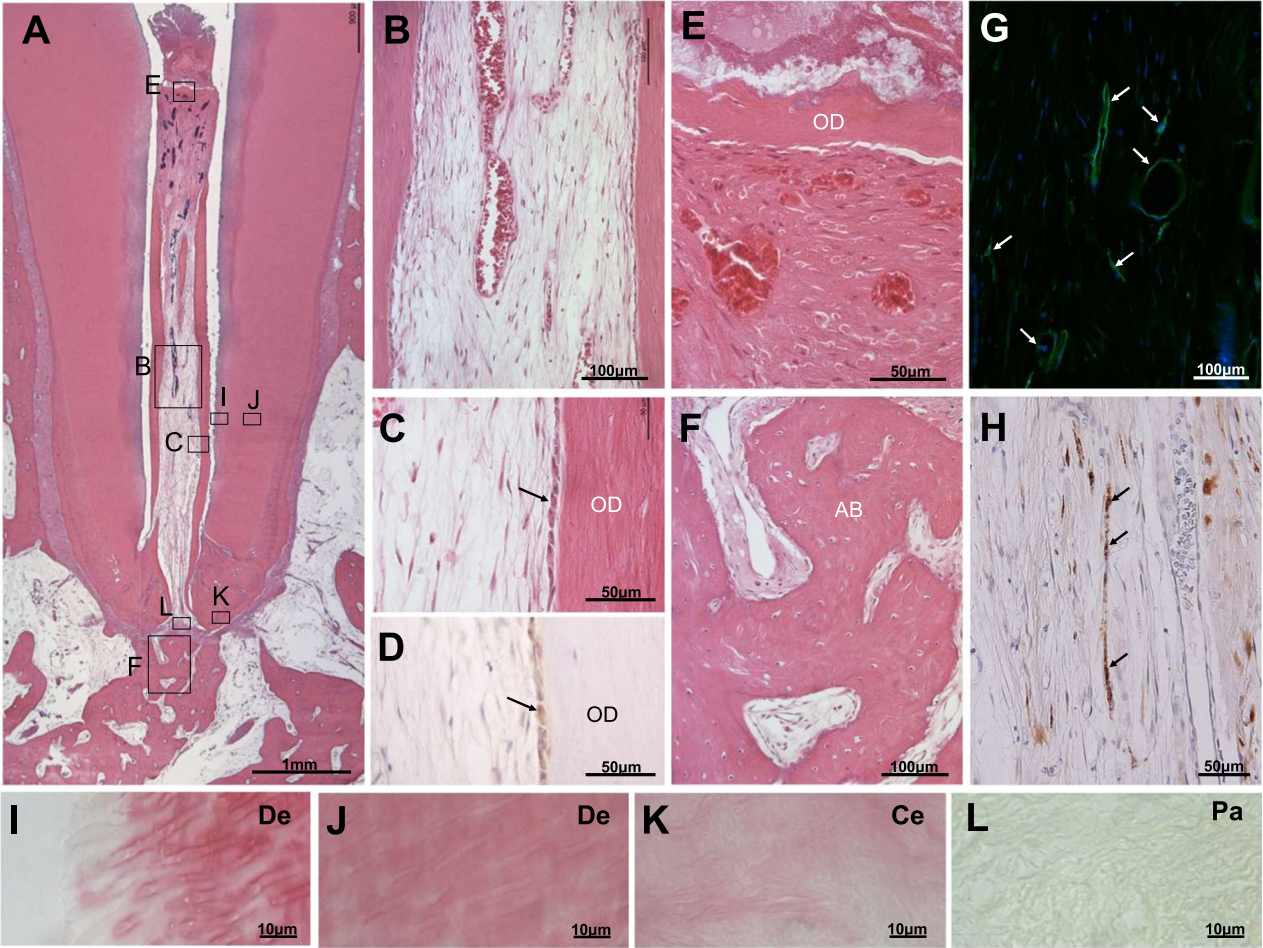
Pulp regeneration after complete disinfection with levofloxacin and nanobubbles in model A. **A–C**, **E**, **F** Hematoxylin and eosin staining. **A**, **B** Regenerated pulp tissue. **C**, **D** Odontoblastic cells (arrow) lining to newly formed osteodentin/tubular dentin (OD) along with the dentin. **D** Immunohistological staining for Dspp. **E** Osteodentin (OD) was regenerated in the coronal part of the root canal. **F** Periapical tissue healing. Alveolar bone (AB). **G** Blood vessels stained by BS-1 lectin (arrows). **H** Neuronal process stained by PGP 9.5 (arrows). **I**–**L** Modification of the Brown and Brenn Gram staining. **I** Root canal surface of dentin (De). **J** Deep inside dentin (De). **K** Cementum (Ce). **L** Periapical tissue (Pa)

In model A, after irrigation and intracanal medication for one month, the number of residual bacteria decreased from 10^3^ to 10^7^ colony-forming units (cfu) to below the detection level in all 12 root canals. Two months after DPSC transplantation, pulp regeneration was observed in 8 out of the 12 root canals (Fig. 2A, B). Dentin-like tissue was formed along the lateral wall of the root canal, with alignment of odontoblast-like cells (Fig. 2C). These odontoblast-like cells stained positive with Dspp antibodies (Fig. 2D). Osteodentin-like mineralized tissue was formed on the top of the regenerated pulp tissue (Fig. 2A, E). Revascularization (Fig. 2G) and neurite extension (Fig. 2H) were also observed in the regenerated pulp. Little infiltration of inflammatory cells was observed in the regenerated pulp (Fig. 2A, B) and periapical tissue (Fig. 2A, F, Table 1). Gram staining revealed no bacteria on the surface of dentin (Fig. 2I), in the dentinal tubules (Fig. 2J, Table 1), cementum (Fig. 2K) and periapical tissue (Fig. 2L, Table 1).

**Table 1:** Difference in residual bacteria, inflammation, and resorption between the success group and the failure group in model A and model B

	Model A			Model B		
	Regenerated pulp tissues			Regenerated pulp tissues		
	Success (*n* = 8)	Failure (*n* = 4)	*P* value	Success (*n* = 10)	Failure (*n* = 5)	*P* value
Residual bacteria						
Intracanal	0.0 ± 0.0	0.5 ± 0.5	0.091	0.3 ± 0.5	0.6 ± 0.5	0.166
Periapical	0.0 ± 0.0	0.8 ± 0.4	0.029*	0.4 ± 0.5	1.0 ± 0.0	0.003**
Inflammation score (DAIS)	0.8 ± 0.7	2.8 ± 1.3	0.034*	0.9 ± 0.8	4.0 ± 0.0	< 0.0001***
Resorption						
Inner Resorption	0.0 ± 0.0	0.3 ± 0.4	0.196	0.1 ± 0.3	0.4 ± 0.5	0.163
Outer Resorption	0.0 ± 0.0	0.8 ± 0.4	0.029*	0.2 ± 0.4	0.2 ± 0.4	0.465

*P < 0.05; **P < 0.01; ***P < 0.001

**Fig. 3:**
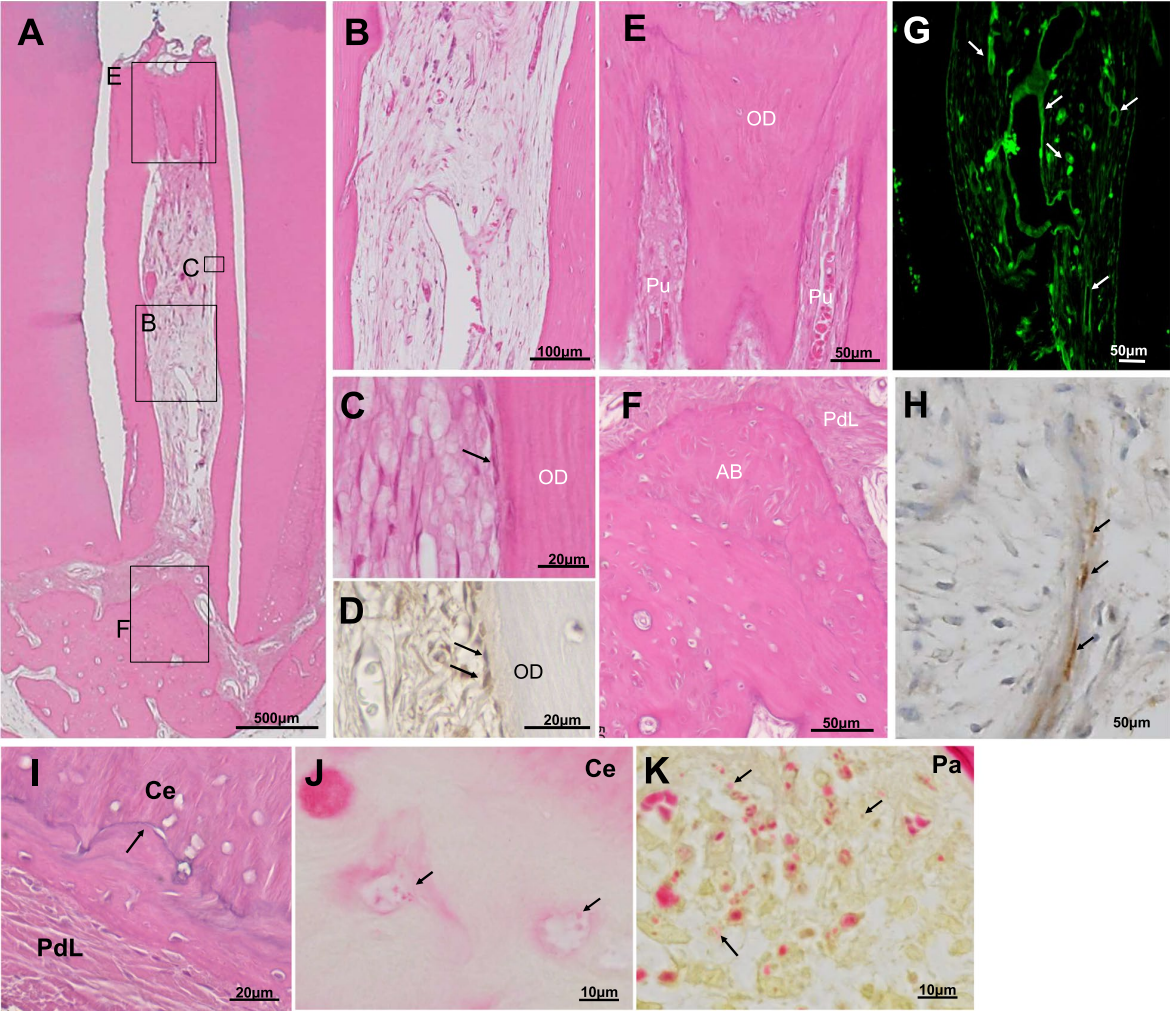
Pulp regeneration after complete disinfection with levofloxacin and nanobubbles in model B. **A**–**C**, **E**, **F**, **I** Hematoxylin and eosin staining. **A**, **B** Regenerated pulp tissue. **C**, **D** Odontoblastic cells (arrows) lining to newly formed osteodentin/tubular dentin (OD) along with the dentin. **D** Immunohistological staining for Dspp. **E** Osteodentin (OD) was regenerated in the coronal part of the root canal. **F** Periapical tissue healing. Alveolar bone (AB). Periodontal ligament (PdL). **G** Blood vessels stained by BS-1 lectin (arrows). **H** Neuronal process stained by PGP 9.5 (arrows). **I** Healing of external resorption (arrow) with formation of cementum (Ce). Periodontal ligament (PdL). **J**, **K** Modification of the Brown and Brenn Gram staining. Gram-negative bacteria (arrows). **J** Cementum (Ce). **K** Periapical tissue (Pa)

In model B, anaerobic bacterial culture showed the number of residual bacteria decreased below the detection level in 12 out of the 15 root canals and the PCR assay showed negative in 11 out of the 15 root canals after the repeated disinfection for 3 months. The number of disinfection was significantly higher in model B compared with that in model A (7.13 ± 2.09 times (*n* = 15); 1.17 ± 0.37 times (*n* = 12), respectively, *P* < 0.0001). Six months after transplantation, pulp regeneration was observed in 10 out of the 15 root canals (Fig. 3A, B). As seen in model A, dentin-like tissue was formed along the dentinal wall (Fig. 3C). The odontoblast-like cells attached to the wall were less in number compared with those in model A, although they stained positive with Dspp antibody (Fig. 3D). Osteodentin-like tissue on the top of the regenerated pulp tissue (Fig. 3A, E), revascularization (Fig. 3G), neurite extension (Fig. 3H), and healed periapical tissue (Fig. 3A, F) were also observed in the regenerated pulp, as shown in model A. The DAIS classification, which indicates the degree of inflammation of the periapical tissue, showed a mild grade 0–2 with an average of 0.9 ± 0.8 (*n* = 10) in the success group in model B, which was not significantly different from the DAIS average of 0.8 ± 0.7 (*n* = 8) in model A (Table 1). In the success group, 6 out of 10 root canals showed periapical tissue repair by cementum formation on the surface of external resorption (Fig. 3I), although mild external resorption was seen in two root canals and internal resorption in one root canal. Gram staining revealed some residual bacteria in the cementum (Fig. 3J) and apical periodontium (Fig. 3K) (Table 1) in 4 out of the 10 root canals, unlike in model A where no bacteria were present (Fig. 2I–L).

In both model A and B, there was a tendency for pulp regeneration to be successful in the absence of bacteria in the periapical tissue and low inflammation score.

### Relationship between relative amount of regenerated pulp and the number of bacteria, lesion volume before treatment and the reduction rate of lesion in the success groups

**Fig. 4:**
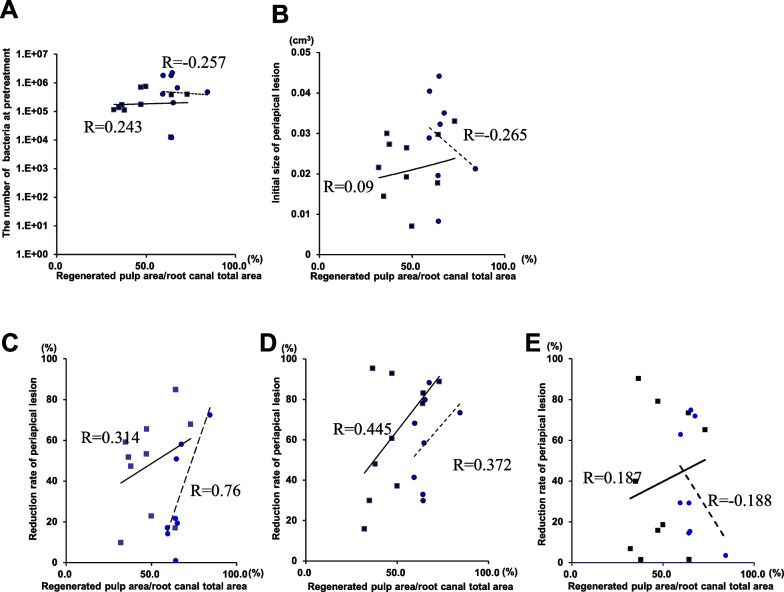
Statistical analyses of correlation of relative amount of regenerated pulp with the bacterial number and lesion volume before root canal treatment, and with reduced rates of lesion volume in the successful root canals. **A** No correlation of relative amount of regenerated pulp with bacterial number before root canal treatment (model A: *R* =  − 0.257, model B: *R* = 0.243). **B** No correlation of relative amount of regenerated pulp with the lesion volume before root canal treatment (model A: *R* =  − 0.265, model B: *R* = 0.090). **C** Correlation of relative amount of regenerated pulp with reduced rates of lesion volume at cell transplantation compared with that before root canal treatment (model A: *R* = 0.314, model B: *R* = 0.760). **D** Correlation of relative amount of regenerated pulp with reduced rates of lesion volume at extraction compared with that at before root canal treatment (model A: *R* = 0.445, model B: *R* = 0.372). **E** No correlation of relative amount of regenerated pulp with reduced rates of lesion volume at extraction compared with that at cell transplantation (model A: *R* = 0.187, model B: *R* =  − 0.188). Model A: round dashed line, model B: square solid line. Eight root canals in model A and 10 root canals in model B with successful pulp regeneration

Next, in the success groups of model A and model B, the relative amount of regenerated pulp was 66.2 ± 7.9% at 2 months in model A and 48.7 ± 14.2% at 6 months in model B (Additional file 1: Table S1). In 8 and 10 root canals of model A and model B, the relative amounts of regenerated pulp were hardly related to the number of bacteria before the start of root canal treatment (*R* =  − 0.257 and *R* = 0.243, respectively) (Fig. 4A). It was also shown that the relative amount of regenerated pulp in the success groups of model A and model B was largely unrelated to the lesion volume prior to root canal treatment (*R* =  − 0.265, and *R* = 0.09, respectively) (Fig. 4B). In both model A and model B, the relative amount of regenerated pulp was correlated with the lesion reduction rate from before treatment to transplantation (Fig. 4C) (*R* = 0.76, and *R* = 0.31, respectively), and with that from before root canal treatment to extraction (Fig. 4D) (*R* = 0.37, and *R* = 0.45, respectively) (Additional file 1: Table S1), indicating that the higher lesion reduction rate at transplantation and at extraction, the higher pulp regeneration rate. On the other hand, there was no correlation between the relative amount of regenerated pulp and the lesion reduction rate from transplantation to extraction (Fig. 4E) (*R* =  − 0.188, and *R* = 0.187, respectively).

### Histological and bacteriological analyses in the failure groups

**Fig. 5:**
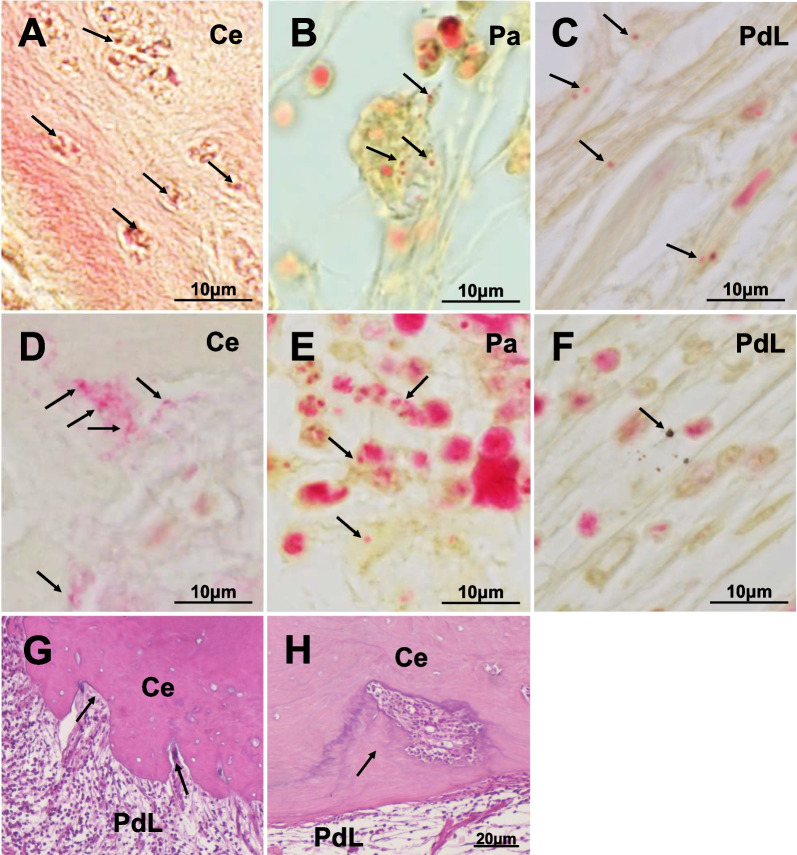
Histobacteriological analyses of root canals with unsuccessful pulp regeneration. **A**–**F** Residual bacteria in the periapical legion detected by gram staining. Gram-negative bacteria (arrows) **G, H** H-E staining. **A**–**C** model A. **D–F** model B. **A, B** Outer root surface of cementum (Ce). **C**, **D** Periapical lesion (Pa). **E**, **F** Periodontal ligament (PdL). **G** External resorption (arrows) in model B. **H** Healing of external resorption with formation of cementum (arrow) in model B

Pulp regeneration was failed in 4 out of 12 root canals in model A and in 5 out of 15 root canals in model B (Table 1). In the failure groups, residual bacteria were found in 3 out of 4 root canals in model A (Fig. 5A–C) and in all 5 root canals in model B (Fig. 5D–F). On the other hand, in the failure groups, the DAIS grades of the periapical tissue in model A and in model B were 2.8 ± 1.3 (*n* = 4), and 4.0 ± 0.0 (*n* = 5), respectively (Table 1). However, there was a significant difference in the DAIS grades between the success group and the failure group in model A (0.8 ± 0.7 (*n* = 8), and 2.8 ± 1.3 (*n* = 4), respectively, *P* = 0.034), and in model B (0.9 ± 0.8 (*n* = 10), and 4.0 ± 0.0 (*n* = 5), respectively, *P* < 0.0001), respectively (Table 1). DAIS grade 2 or higher inflammation were found in 3 out of 4 root canals in model A and in all 5 root canals in model B in the failure groups (Table 1). While in the success group, inflammation was found in 1 out of 8 root canals in model A and in 4 out of 10 root canals in model B. In the root canals with DAIS grade 2 or higher inflammation, residual bacteria were found in 2 out of 4 root canals in model A and in 7 out of 9 root canals in model B, while in the root canals without inflammation, in 1 out of 8 root canals in model A and in 2 out of 6 root canals in model B. Residual bacteria were highly associated with inflammation of the apical periodontium (odds ratio 8.3), indicating that residual bacteria in the apical periodontium may increase the risk of progressive inflammation by 2.8 times (risk ratio 2.8). The multiple logistic regression analysis demonstrated that residual bacteria in the periapical tissue was significantly associated with inhibition of pulp regeneration (OR = 0.075, 95% CI = 0.06–0.994, *P* = 0.049) (Table 2), indicating that the risk of pulp regeneration being compromised by residual bacteria may be tenfold. Inflammation of the periapical tissue was also significantly associated with inhibition of pulp regeneration (OR = 0.075, 95% CI = 0.06–0.994, *P* = 0.049) (Table 2), indicating that the risk of pulp regeneration being compromised by inflammation may be 2.8 times.

**Table 2:** Multiple logistic regression analyses of correlation of presence of regenerated pulp tissue with presence of residual bacteria and inflammation

Variables	OR	95% CI	*P* value
Residual bacteria	0.075	0.060–0.994	0.049*
Inflammation	0.075	0.060–0.994	0.049*

*OR* Odds ratio, *CI* Confidence interval

*P < 0.05

Furthermore, no internal or external resorption was observed in the success group and in the failure group in model A (Table 1). One out of 5 root canals in model B had external resorption (Fig. 5G) and 4 root canals had repaired external resorption (Fig. 5H).

Therefore, these results suggest that residual bacteria in the periapical tissue may exacerbate inflammation and inhibit pulpal regeneration.

### The cytokinetics of transplanted cells in infected teeth

**Fig. 6:**
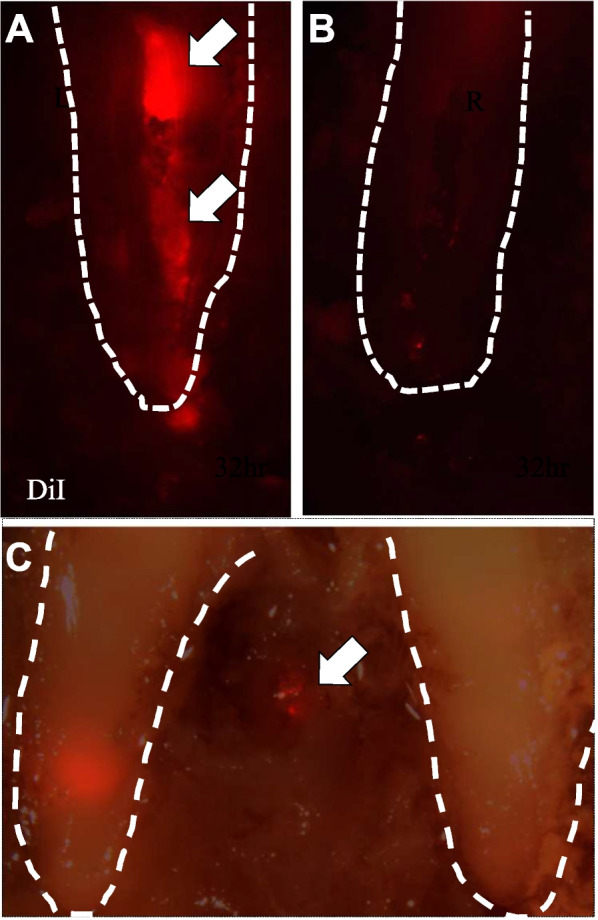
Migration analysis of DiI-labelled dental pulp stem cells (DPSCs) 72 h after transplantation in the apical periodontitis model. Outline of the teeth are indicated by dotted lines. **A** Transplantation into the root canals after complete disinfection. The transplanted DPSCs (red) localizing inside the root canals (arrows). **B**, **C** Transplantation without root canal treatment. Few transplanted DPSCs in the infected root canals. **C** Note the DPSCs migrating out from the canal to fistula (arrow)

To investigate microenvironmental factors that did not yield satisfactory results in cell transplantation, the cytokinetics of transplanted cells in infected teeth without root canal treatment and in infected teeth with complete disinfection were compared. At 72 h after transplantation, the transplanted cells remained in the disinfected root canal (Fig. 6A). However, few graft cells were detected in the infected root canal (Fig. 6B) and some graft cells were observed around the fistula, suggesting that graft cells migrated from the root canal toward the fistula (Fig. 6C).

## Discussion

### Apical periodontitis models with moderate and severe infection in dogs

In this study, we investigated the effect of residual bacteria in the root canal and the periapical tissue on the outcome of pulpal regeneration by pulp regenerative cell therapy for mature teeth with periapical lesions. First, two distinct canine models of apical periodontitis, model A (moderate infection) and model B (severe infection), were constructed by changing the duration of canal exposure to the oral cavity and closure. Cardoso et al. [24] reported that bacterial number correlate with the size of the root apex lesion, and we found a similar correlation between them. There was no difference in bacterial number and apical lesion volumes between model A and model B before starting root canal treatment. However, anaerobic bacteria, *Tissierella sp*, *Fusobacterium sp*, and facultative anaerobes, *Lactobacillus murinus* and *Escherichia coli strai* were identified in the root canals of model A. These bacteria are frequently detected from permanent teeth with the primary root canal infection [25, 26]. While, model B had the different microbial profile from model A: facultative anaerobes, such as *Enterococcus faecalis* and *Tessaracoccus massiliensis*, and obligate anaerobes, *Porphyromonas sp*, *Prevotella sp*, and *Tannerella forsythia* were identified. These bacteria are identified in persistent apical periodontitis to contribute in etiology of periapical infection [27]. Swelling and fistulas were observed in 6 out of the 15 root canals in model B, and external resorption by CBCT image was also detected in 10 out of the 15 canals, indicating more microbial invasion into the complex root canal system and the root canal infection more leaching through the apical foramen in model B than in model A. Inflammation in the periapical tissue might also be worse in model B than in model A. Therefore, it was suggested that complete disinfection might be more difficult in model B. In fact, root canal treatment using levofloxacin-containing nanobubble was repeated more in model B (about 7 times for 3 months) compared with that in model A (about 1 time for 1 month). While, levofloxacin exhibits antibacterial activity against most of the bacteria isolated from the root canals in both model A and model B. Thus, these differences in disinfection frequency and duration may be due to infections in the deeper inaccessible sites within the root canal systems and periapical tissues. The number of bacteria decreased below the detection level in all 12 root canals in model A. While, in model B, the number of bacteria not decreased below the detection level in 3 out of 15 root canals, and PCR tests were positive for bacteria in 4 root canals, indicating that approximately 27% of the root canals was failure in complete disinfection before cell transplantation in model B. Thus, the two distinct models for apical periodontitis could be established to study the effects of residual bacteria and inflammation status in the periapical tissue on pulpal regeneration.

### Root canals with successful pulp regeneration

The clinical trial on pulp regenerative cell therapy have been performed in human mature teeth with apical periodontitis [11]. However, the outcome of pulp regeneration was only assessed by a positive response to electric and cold tests, a remission of periapical lesions, and an increase in the perfusion unit percentage by laser Doppler flowmetry. The formation of a vascularized tissue with a normal physiological function, probably vital pulp-like tissue, was indicated, but there was no histological finding to demonstrate regeneration of the pulp-dentin complex due to ethical issues. In a ferret apical periodontitis model in immature teeth, the presence of loose connective tissue in conjunction with osteodentin in the root canal has been reported three months after pulp regenerative therapy with DPSCs [12]. In the present study, histological analysis demonstrated that the successful group showing 30% or more relative amount of regenerative pulp was accounted for approximately 67%, that is 8 out of the 12 root canals in model A and 10 out of 15 root canals in model B. In both model A and model B, the true pulp regeneration was similarly observed as reported in the cell therapy for irreversible pulpitis [22]; including loose connective tissue with revascularization and reinnervation, dentin-like mineralized tissue along the lateral dentinal wall with alignment of odontoblast-like cells, and osteodentin-like mineralized tissue on the regenerated pulp in the upper part of the root canal. Periapical tissues were healed and inflammation was a little (in model A) or mild (in model B). In model B, the external resorption was repaired by cementum formation in 6 out of 10 root canals. The relative amount of pulp regeneration was higher in model A compared with that in model B (66.2% at 2 months, and 48.7% at 6 months, respectively). Therefore, there was a not qualitative but quantitative difference in the successful pulp regeneration between the two models. On the other hand, the residual bacteria in the periapical tissue of model B (0.4) was significantly higher than that of model A (0), while there was no significant difference in DAIS inflammation grade. Therefore, the quantitative difference between model A and model B part may be partly due to residual bacteria in the periapical tissue. Furthermore, as pointed out by Kim [18], repeated irrigation with 6% sodium hypochlorite might be responsible for the quantitative difference, since resident stem cells were damaged and their migration into the root canal were inhibited.

### Root canals with unsuccessful pulp regeneration

In a ferret apical periodontitis model in immature teeth, the presence of residual bacteria was correlated with a lack of radiographic root development and reduced amount of dentin-associated mineralized tissue formation, indicating a major drawback to the successful pulp regeneration [12]. In a mini-swine apical periodontitis model established by filling the canals with periodontal plaque, after sodium hypochlorite irrigation and intracanal medication using triple antibiotic paste, periapical bone healing with minimal apical radiolucency was confirmed in 5 weeks and DPSC transplantation was performed. However, recurrence of apical lesions, inflammation in the root canal with apical resorption and heavily infected dentinal tubules were reported after 8 weeks [14]. In the present study, the pulp regeneration failure groups, in which the relative amount of regenerated pulp was less than 30%, were accounted for approximately 33% (4 out of 12 root canals in model A and 5 of 15 root canals in model B, despite of irrigation and intracanal medication with nanobubbles containing levofloxacin. Residual bacteria were found in 3 of 4 root canals in model A and all 5 root canals in model B in the failure groups. The DAIS grade of inflammation in the periapical tissue was significantly different not between the two models, but between the successful and failure groups (< 0.05, and < 0.0001 for model A and model B, respectively). Multiple logistic regression analysis also showed that residual bacteria and inflammation of periapical tissue were associated with failure pulpal regeneration (OR = 0.075). Thus, our results suggest that residual bacteria in the periapical tissue may exacerbate inflammation and inhibit pulp regeneration.

### Confirmation of aseptic conditions

In the clinical study by Brizuela et al. [11] of pulp regenerative cell therapy in mature teeth with apical lesions, the root canals were disinfected with 2.5% sodium hypochlorite, sonic activated with the Endoactivator system, and treated with calcium hydroxide for 3 weeks. The canal was irrigated with 17% EDTA to remove the calcium hydroxide before cell transplantation. However, there is no mention of confirmation of aseptic conditions in the root canal. In the case report by Gomez-Sosa et al. [28], the tooth did not show any evidence of infection after 5.25% sodium hypochlorite irrigation and intracanal medication with calcium hydroxide for 3 weeks, and then further irrigation with 17% EDTA. However, it is not clearly elucidated how to confirm no evidence of infection. In the present study, in the failure groups the number of bacteria could not decrease below the detection level by anaerobic bacterial culture in none of 4 root canals in model A, and in 3 out of 5 root canals in model B. While 4 out of 5 root canals in model B tested positive for the PCR assay. However, histobacteriological analyses in the failure groups detected residual bacteria in 3 out of 4 root canals in model A and in all 5 root canals in model B. Therefore, based on these results, it is difficult to confirm aseptic condition only by the anaerobic bacterial culture method as in model A. In some cases, as in model B, the PCR assay may have the advantage of detecting bacteria that are as-yet-uncultivable or difficult-to-cultivate. The PCR assay therefore may possibly complement conventional microbiological diagnostics [20]. There was one root canal in model B which tested negative both for the anaerobic bacterial culture method and the PCR assay. Thus, in order to increase the success rate of pulp regeneration, it is essential to develop a more sensitive protocol especially for harvesting microorganisms from biofilms in the periapical tissue in addition to for confirming aseptic conditions.

### Correlation between reduced lesion volume and relative amount of regenerative pulp

In this study, there was no correlation between the lesion volume before starting root canal treatment and the relative amount of regenerated pulp in the successful groups in model A and model B. Thus, it was suggested that pulp regeneration is possible even when apical lesion volume is large. In the successful groups, the relative amount of regenerated pulp was associated with the reduced rate of lesion volume from before root canal treatment to transplantation and from before root canal treatment to extraction. Thus, the reduced rate of lesion volumes from root canal treatment to transplantation and to extraction may be potential indicators to determine the optimal timing of cell transplantation and the outcome of pulp regeneration, respectively.

### Negative effects of residual bacteria and inflammation on pulp regeneration

Furthermore, we examined the negative effects of residual bacteria on pulp regeneration. Transplanted cells were absent from the root canals that were not disinfected, and some of these cells migrated to the fistula. Bacterial biofilms that develop outside the apical foramina are associated with refractory periapical periodontitis [29]. Inflammatory cytokines are released from immune cells in the surrounding periapical tissues in the presence of biofilm outside the apical foramen [30]. Increased concentrations of inflammatory cytokine, especially CXCL12 in the local inflammatory site, are critical signals for mesenchymal stem cell migration [31]. We previously reported that DPSCs have high levels of CXCR4, which is a receptor for CXCL12 [22]. Thus, transplanted DPSCs may have migrated out of the apical foramen via the CXCR4-CXCL12 axis in the presence of a biofilm, resulting in no pulp tissue regeneration in the root canal. Additional procedures are needed to prevent DPSC migration outside the root canal for pulp regenerative cell therapy for apical periodontitis.

## Conclusions

In conclusions, the pulp regenerative therapy using allogeneic DPSCs in mature teeth with apical lesions was performed in canine models, and histological analyses revealed that a true pulp-dentin complex was regenerated in the root canal. Furthermore, morphometric and histobacteriological analyses revealed negative associations of successful pulp regeneration both with residual bacteria and inflammation in the periapical tissue.

### Supplementary Information

**Additional file 1: Table S1.** Difference in relative amount of regenerated pulp, periapical lesion volumes and reduced rates of lesion volume between pulp regenerated and not regenerated root canals in model A and model B.

## Data Availability

All data generated or analyzed during this study are included in this published article.

## Abbreviations

DPSCs: Dental pulp stem cell
CB-RET: Cell-based regenerative endodontics
CF-RET: Cell-free regenerative endodontic therapy
EPT: Electric pulp testing
MRI: Magnetic Resonance Imaging
MSs: Mesenchymal stem cells
CBCT: Cone-beam computed tomographer
BHI: Brain heart infusion
PCR: Polymerase Chain Reaction
EDTA: Ethylenediaminetetraacetic acid
G-CSF: Granulocyte colony-stimulating factor
DSPP: Dentin sialophosphoprotein
DAIS: Dental Apical Inflammation Score
CXCR4: C-X-C motif chemokine receptor 4
CXCL12: C-X-C motif chemokine ligand 12
