# Supplementary material for: Periapical bacterial disinfection is critical for dental pulp regenerative cell therapy in apical periodontitis in dogs
Source: Stem Cell Res Ther. 2024 Jan 17;15:17. doi: 10.1186/s13287-023-03628-6 (PMC10792888; doi:10.1186/s13287-023-03628-6)
Supplement: Supplementary file 1 — Additional file 1: Table S1. Difference in relative amount of regenerated pulp, periapical lesion volumes and reduced rates of lesion volume between pulp regenerated and not regenerated root canals in model A and model B. [file 13287_2023_3628_MOESM1_ESM.docx]

|  | model A | | | model B | | |
| --- | --- | --- | --- | --- | --- | --- |
|  | regenerated pulp tissues  (2 months) | | | regenerated pulp tissues  (6 months) | | |
|  | Success (*n* = 8) | Failure (*n* = 4) | *P* value | Success (*n* = 10) | Failure (*n* = 5) | *P* value |
| Regenerated pulp volume  /total root canal volume（％） | 66.2 ± 7.9 | 1.0 ± 2.1 | <0.0001 | 48.7 ± 14.2 | 5.8 ± 5.3 | <0.0001 |
| PA volume (mm^3^) |  |  |  |  |  |  |
| before treatment | 28.7 ± 11.9 | 18.7 ± 13.1 | 0.584 | 25.3 ± 9.4 | 23.1 ± 12.0 | 0.389 |
| transplantation | 18.8 ± 9.6 | 14.3 ± 10.2 | 0.876 | 11.8 ± 4.9 | 11.2 ± 9.0 | 0.254 |
| extraction | 10.4 ± 5.6 | 5.1 ± 3.5 | 0.061 | 7.0 ± 5.6 | 7.4 ± 6.3 | 0.554 |
| Reduced rate of PA volume (%) |  |  |  |  |  |  |
| transplantation / before treatment | 31.7 ± 25.2 | 18.0 ± 15.9 | 0.295 | 47.9 ± 24.2 | 57.0 ± 19.3 | 0.622 |
| extraction / before treatment | 58.9 ± 22.1 | 66.1 ± 26.9 | 0.897 | 67.7 ± 26.6 | 72.6 ± 11.1 | 0.069 |
| extraction / transplantation | 37.6 ± 28.1 | 56.1 ± 32.6 | 0.543 | 39.2 ± 34.9 | 30.6 ± 23.4 | 0.099 |

Supplemental Table

Difference in relative amount of regenerated pulp, periapical lesion volumes and reduced rates of lesion volume between the success group and the failure group

PA: Periapical lesion
